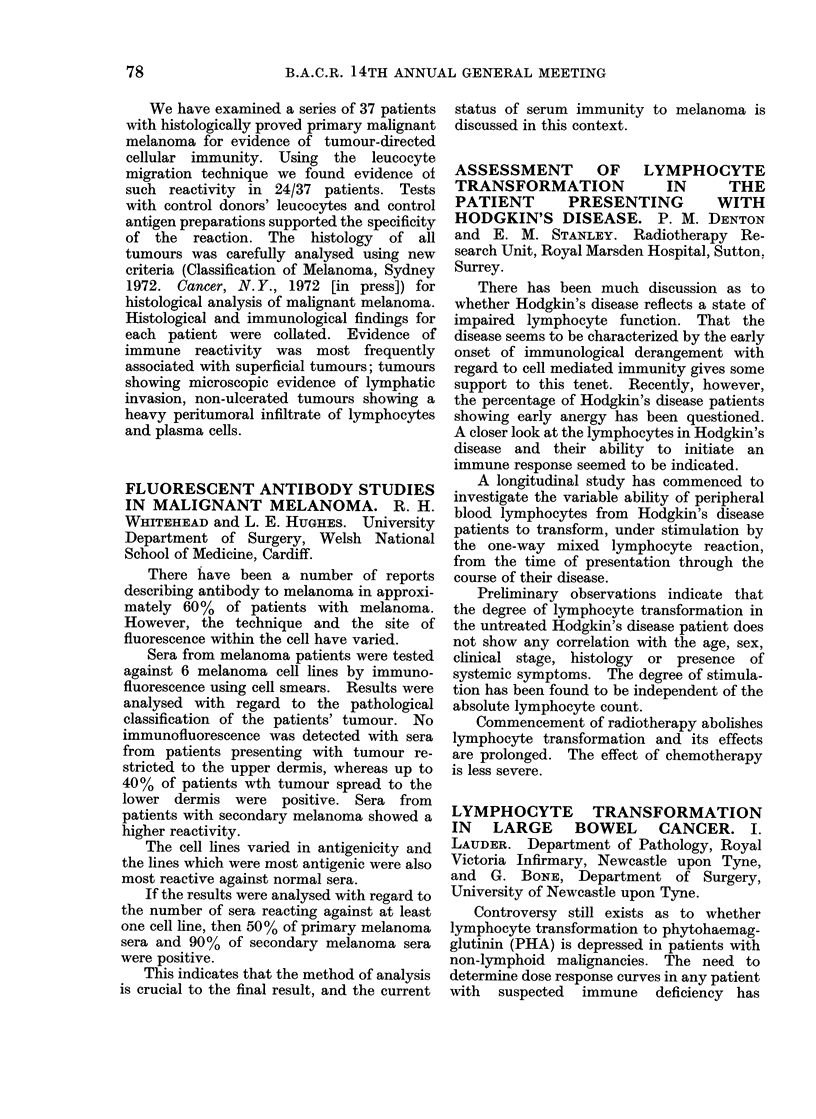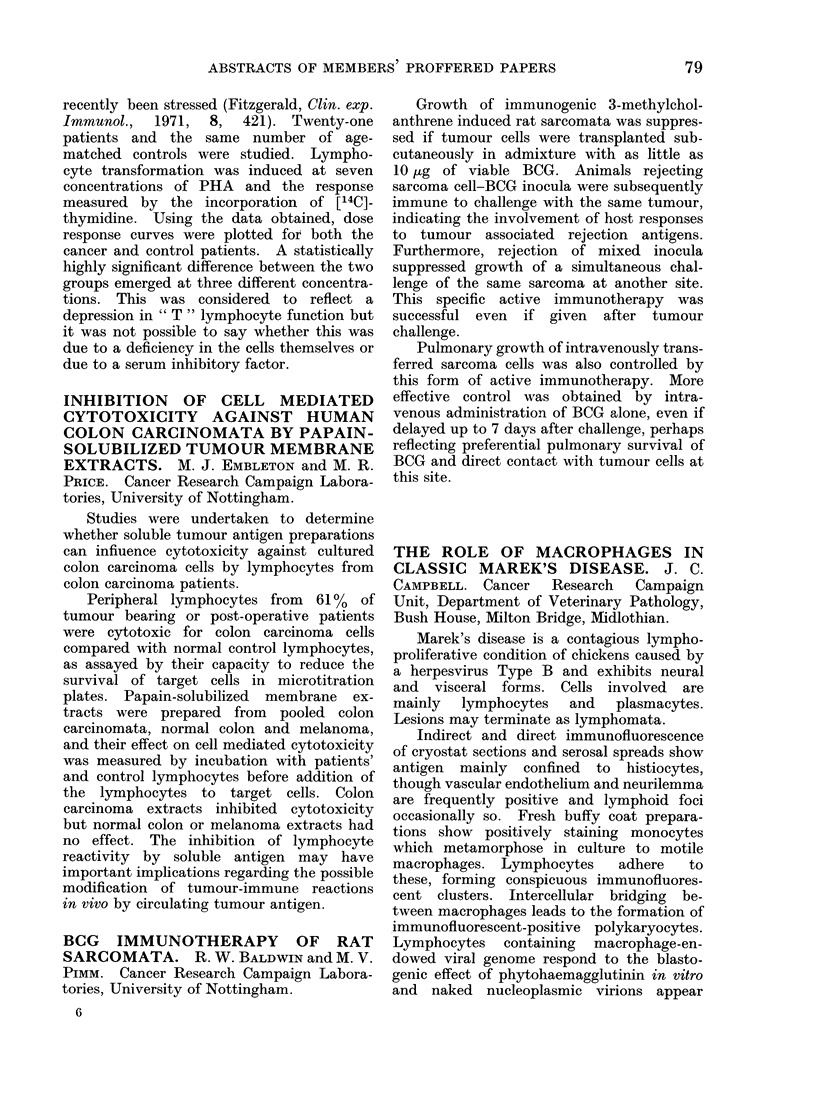# Lymphocyte transformation in large bowel cancer.

**DOI:** 10.1038/bjc.1973.82

**Published:** 1973-07

**Authors:** I. Lauder, G. Bone


**LYMPHOCYTE TRANSFORMATION IN LARGE BOWEL CANCER.** I. Lauder. Department of Pathology, Royal Victoria Infirmary, Newcastle upon Tyne, and G. Bone, Department of Surgery, University of Newcastle upon Tyne.

Controversy still exists as to whether lymphocyte transformation to phytohaemagglutinin (PHA) is depressed in patients with non-lymphoid malignancies. The need to determine dose response curves in any patient with suspected immune deficiency has recently been stressed (Fitzgerald, *Clin. exp. Immunol.*, 1971, **8**, 421). Twenty-one patients and the same number of age-matched controls were studied. Lymphocyte transformation was induced at seven concentrations of PHA and the response measured by the incorporation of [$^{14}$C]-thymidine. Using the data obtained, dose response curves were plotted for both the cancer and control patients. A statistically highly significant difference between the two groups emerged at three different concentrations. This was considered to reflect a depression in " T " lymphocyte function but it was not possible to say whether this was due to a deficiency in the cells themselves or due to a serum inhibitory factor.